# Examining the unsustainable relationship between SDG performance, ecological footprint and international spillovers

**DOI:** 10.1038/s41598-024-61530-4

**Published:** 2024-05-17

**Authors:** Mustafa Moinuddin, Simon Høiberg Olsen

**Affiliations:** Institute for Global Environmental Strategies, Hayama, Japan

**Keywords:** Environmental impact, Sustainability

## Abstract

For almost a decade, countries have been working to achieve the Sustainable Development Goals (SDGs). Yet progress on the SDGs across countries, as well as across the 17 goals, has proven frustratingly slow. Even countries that have performed relatively well on the SDGs may have done so by causing negative externalities, such as environmental degradation, in other parts of the world. To determine if this is the case empirically, we developed and tested hypotheses concerning how a country’s SDG performance is associated with such externalities. We then ran a regression to examine correlations between indices measuring SDG progress, ecological footprints and international spillovers. We found that SDG progress is positively correlated with increased ecological footprints and spillovers. The results indicate that SDG progress remains closely associated with conventional measures of economic growth, and that negative environmental and social impacts of internationally-sourced consumption represent behavioural and structural barriers to meaningful progress on sustainability.

## Introduction

**Figure 1:**
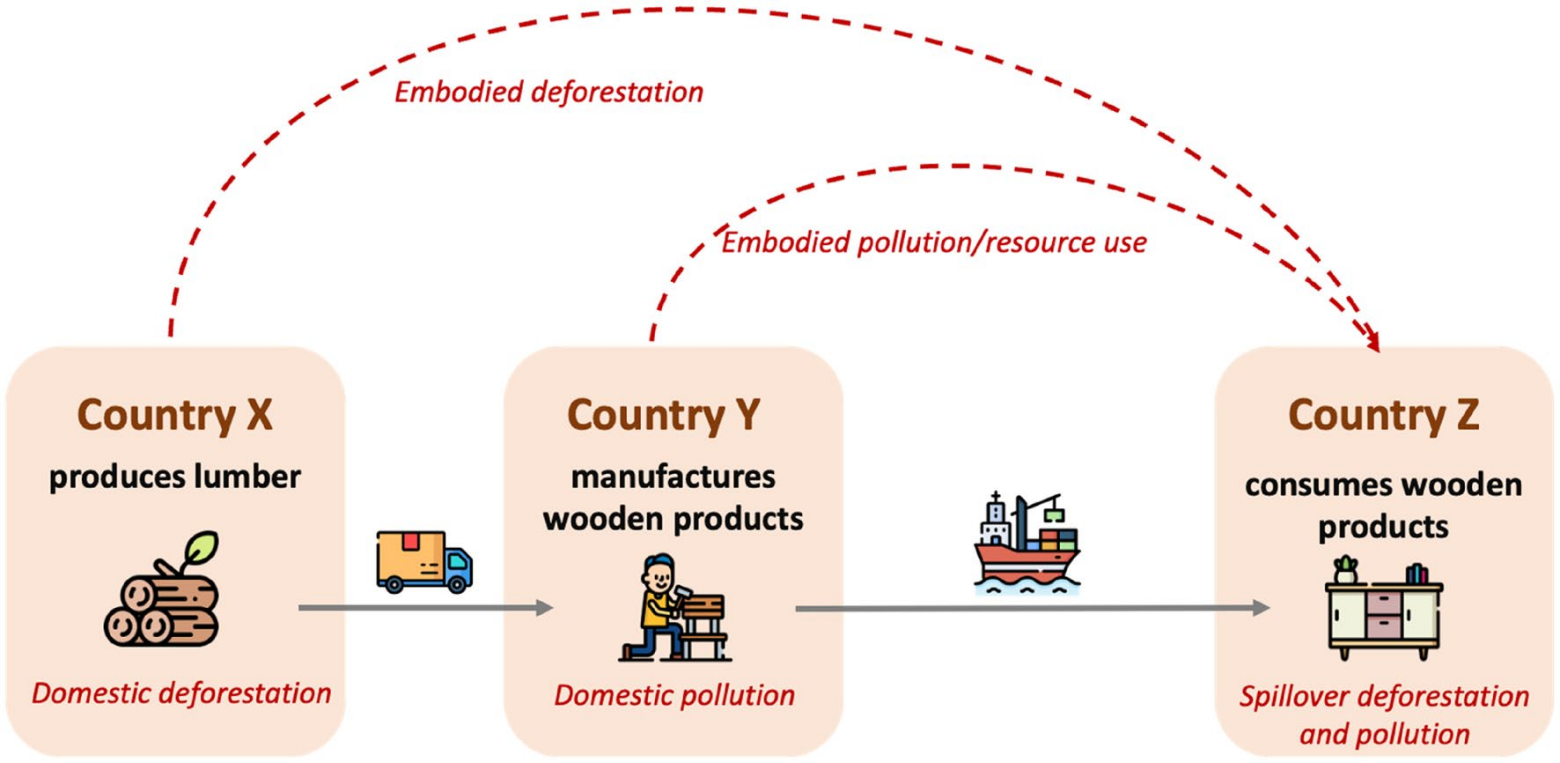
Illustrative example of how international spillovers occur. Source: Adapted from SDG Transformation Center^14^.

The world is not on track to achieve the Sustainable Development Goals (SDGs)^1,2^. As of 2023, a mere 12% of SDGs are likely to be achieved in 2030^3^, with some estimates suggesting that the world might only achieve the 17 goals in 2073^4^. Despite these bleak prospects, some countries are making more progress on the SDGs than others. The Sustainable Development Solutions Network (SDSN) and Bertelsmann Foundation publish an annual SDG Index to track countries’ progress on the SDGs. The SDG Index scores indicate that higher-income countries tend to perform better on the SDGs than lower-income countries. Unfortunately, this performance in richer countries seems to be partly based on externalising social and environmental expenses to other areas in the world (Fig. 1).

The international links between affluence, consumption and the environment represent a growing area of research. Recent work on these themes include studies looking at the international dimensions of biodiversity impacts resulting from land-use change to meet international consumption demands^5^, as well as deforestation caused by international trade in building materials^6^. Concepts that have been developed to reflect this externalization include ‘virtual water’ footprints embedded in trade^7^ or the total associated amount of material extracted to create a product or service or ‘ecological rucksack’^8^. These concepts relate to aspects of the material footprint, which is defined as “the global allocation of used raw material extraction to the final demand of an economy”^9^. To some extent, studies in this area have helped to show that developed and emerging economies have ‘decoupled’ development by shifting away from dependency on domestic resources and relying instead on international trade. However, given that trade also consumes resources, this trend not only causes a displacement of resource use and impacts between production and consumption, but may also increase non-domestic resources relative to the actual quantity of traded goods—a phenomenon captured by the material footprint. Often this trend is not driven directly by countries but by companies engaged in “environmental offshoring”^10,11^, referring to companies relocating resource-intensive parts of their production to developing countries^12,13^. Figure 1 below illustrates this relationship between countries and impacts for the forestry sector.

**Figure 2:**
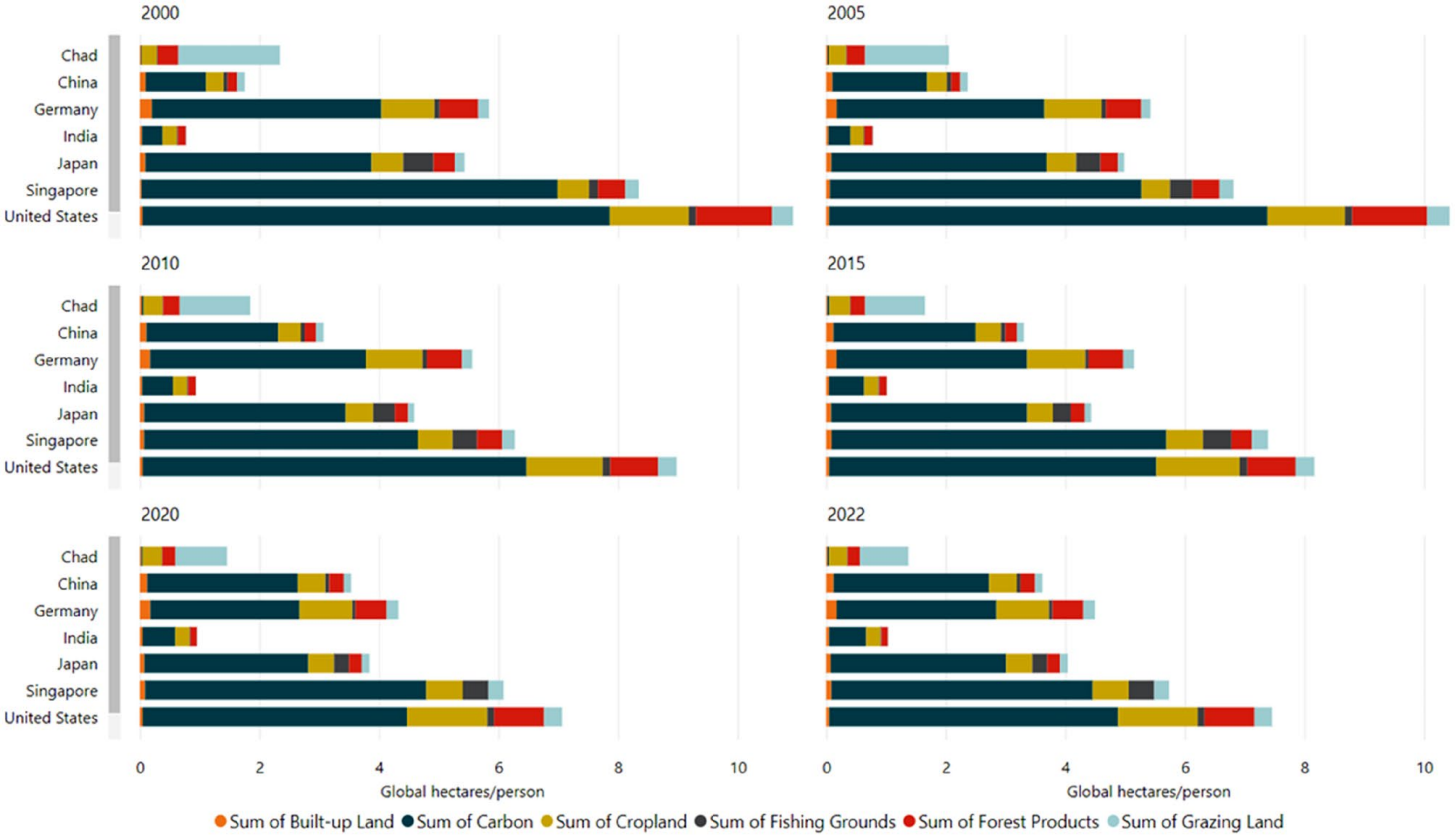
Patterns of ecological footprint of consumption over the years for selected countries. Source: Authors (Based on data from Global Footprint Network^18^).

The internationalization of costs and benefits also impacts the achievement of the SDGs, and the material footprint has been incorporated into SDG indicators on resource efficiency and sustainable management of natural resources^15^. SDSN also examines international material flows in its annual SDG Index reports, and uses the term “spillovers” to connote when one country’s actions generate *benefits* or *costs* on another country that are not reflected in market prices, and therefore are not internalized^16^. Environmental spillovers can include anthropogenic climate change, transboundary pollution and pollution embodied in trade, biodiversity loss embedded in trade, to the misuse of the global commons, such as over-fishing in the high seas^16^. The environmental aspects of SDSN’s Spillover Index also share similarities with the ecological footprint both thematically (Table 1 below) and in terms of how the distribution of the footprint is correlated with development (Fig. 2, also below). The ecological footprint, which measures “…the area of biologically productive land and water that a population (an individual, a city, a country or all of humanity) uses to generate the resources it consumes and absorb its wastes under prevailing technolog(ies)”^17^ has also been used to illustrate the imbalance between resource endowment and final consumption of natural resources across countries. As is evident from Fig. 2, high-income countries typically have a larger ecological footprint.

**Table 1:** Comparing ecological footprint and spillover index. Source: Authors.

Ecological footprint	Spillover Index
Includes domestic and international dimensions	Measures three dimensions: environmental and social impacts embodied into trade, economy and finance, and security
Distinction between domestic and international impacts is unclear	
Usefulness for measuring international impacts is limited	Usefulness for measuring correlation between SDG performance and international impact is clear
Usefulness for measuring correlation between SDG performance and negative environmental impact is clear

Table 1 provides a comparison of the key features of ecological footprint and the Spillover Index. Despite some similarities, the two metrics are distinct in several ways, including their sectoral coverage and international impacts.

While there have been several studies ^5,19–22^ showing a correlation between development, spillovers, and environmental impacts this paper contributes to the literature by providing empirical evidence on the links between SDG performance, ecological footprint, spillovers and conventional measures of development. The added value of such research is clear and compelling: our study shows a positive correlation between national wealth (expressed in GDP per capita, measured in purchasing power parity [PPP] terms) and SDG performance. It also suggests that SDG performance is associated with an increased domestic and international ecological footprint. In this way, we draw attention to international impacts of development, including issues with allocating responsibility, putting appropriate governance in place, and measuring progress toward a global sustainability agenda.

Making headway on poverty, hunger and education is contingent on economic development but if the environment is degraded, eventually the base for sustained prosperity and well-being will also be impacted negatively. With this paper we contribute to the discussion by testing the relationship between SDG progress, ecological footprint and spillovers under the following three hypotheses.

### H1

The richer the country, the better its SDG performance.

### H2

The better a country’s SDG performance, the higher its ecological footprint.

### H3

The richer the country, the worse its spillover score.

## Results

To examine the relationships between SDG performance and the international dimension of associated environmental footprints, we looked at SDSN’s Spillover Index^24^ and the Global Footprint Network’s ecological footprint^25^. Ecological footprint is useful to highlight the environmental impact of a country’s consumption. Although the international dimension is included in the calculation of the aggregate ecological footprint, it does not clearly distinguish the international dimensions of consumption, which means that it is necessary to also look at the Spillover Index. We refer in this paper to either the Spillover Index as the value or to negative spillovers as an impact. By including both these indicators in the empirical checks of hypotheses, both the environmental aspect and the international aspect were captured and examined. To that end, we ran a regression analysis to compare the SDG performance of 163 countries with both their Spillover Index score and their ecological footprint.

The results of the analysis, summarised in Supplementary Information 1, are found to be consistent with our hypotheses. The demographic variable i.e. total population, shows a negative sign, indicating that population size may adversely affect SDG performance. However, the result is not statistically significant. The variable on GDP per capita (in purchasing power parity terms) shows the expected positive sign and high statistical significance (1% level). The coefficient value of 1.17 suggests that a 1% increase in per capita GDP of a country may boost its SDG performance by 1.17%. Trade engagement (represented by total exports) shows statistical significance (5% level) and is also positively associated with SDG performance although the effect is not very strong (coeff. 0.31). The variable for ecological footprint (coeff. 1.55) shows the expected positive impact on SDG performance with statistical significance at the 1% level. The other core explanatory variable, i.e. Spillover Index, is also found to have the expected negative impact on SDG performance, but the coefficient value is very small (coeff. 0.01), with statistical significance at the 5% level.

The results of the dummy variables generate the expected signs and high coefficient values. The prosperity (OECD) dummy has a coefficient value of 0.63, indicating that while membership of the OECD is positively associated with SDG performance, statistical significance is low. This is not surprising, given that the OECD consists of countries that are already among the most developed in the world. This also reinforces our first hypothesis (H1): richer countries tend to perform better on the SDGs. The opposite is seen for LDCs, with these poorer countries still lagging behind in their developmental efforts. Moreover, trade engagement was found to positively impact SDG performance. On the flip side, the variable on total population showed a negative correlation, implying that for many countries, especially developing ones, the size of the population could overburden developmental efforts. The expected negative sign and high coefficient value (coeff. − 9.98) indicate one of the reasons for LDCs’ lower SDG performance. The impact of the diversity (G20) dummy (coeff. 1.08) is positive, which is also not surprising given that G20 countries include both advanced economies and some major developing countries. In brief, the results of our analysis are consistent with our initial hypotheses. See Supplementary Information 1 for the panel regression results.

**Figure 3:**
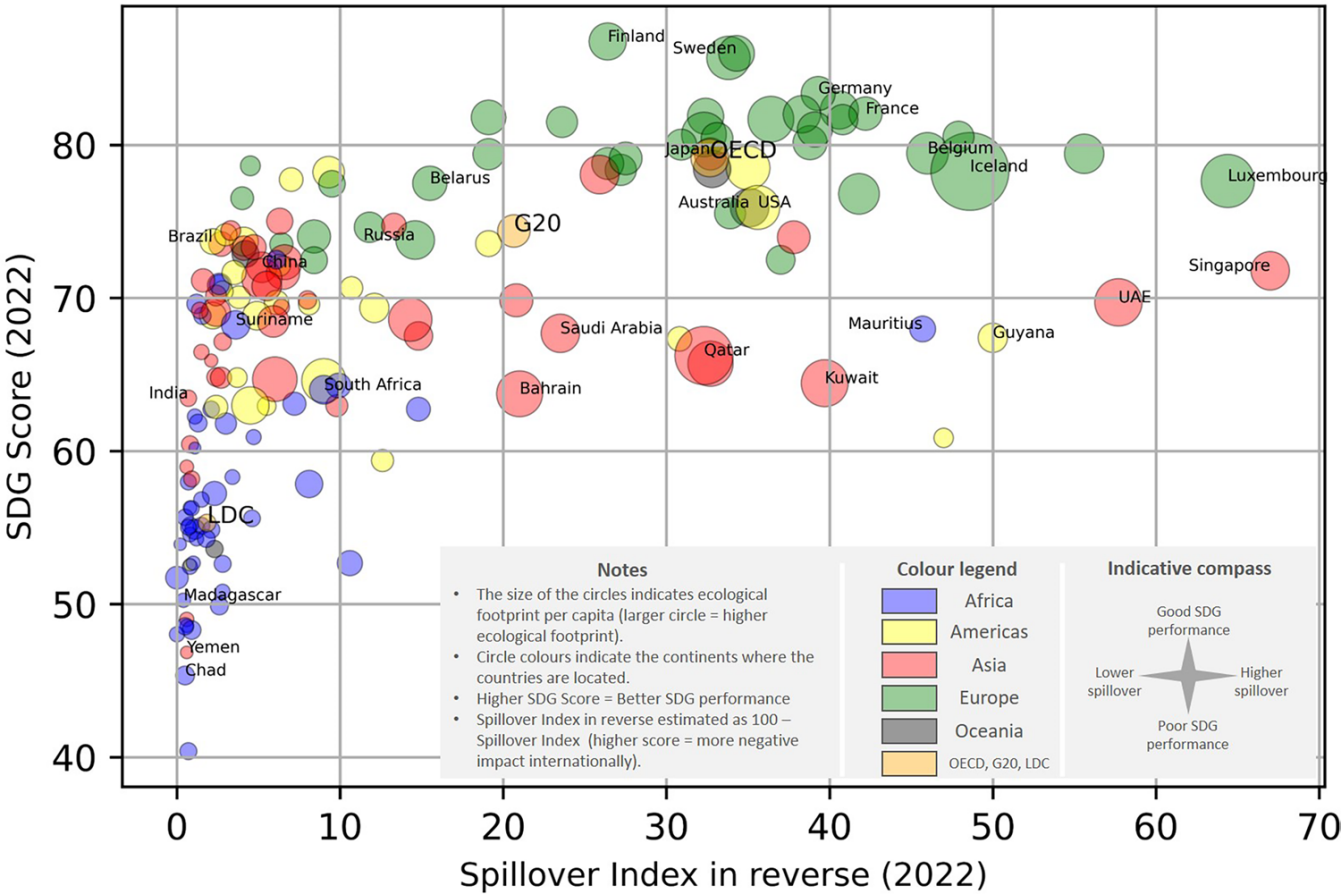
Illustration of the relationship between SDG performance, spillover effect and ecological footprint in 2022. Explanation of the figure: Each bubble is positioned to reflect the respective country’s (or country group’s) SDG performance (SDG Score), and international spillover effect (Spillover Index in reverse), while the size of the bubble indicates that country’s (group’s) ecological footprint. Note that the Spillover Index scores are reversed (100—Spillover Index) so that higher values denote a more negative impact internationally. Furthermore, the colour of the bubbles represents the geographic location of each country. Orange is used for the three groups of countries (OECD, G20, and LDCs). For the country groups, the arithmetic mean is used as an average score. However, our dataset consists of 163 countries in total. Some countries were excluded due to data unavailability. The estimated average score, particularly for the LDC country group, only considers countries that are included in our list of 163 countries. Source: Authors, based on data from SDSN^23^ and Global Footprint Network^18^.

The thematic and geographical interrelationship between performance and impacts can also be visually illustrated with a simple scatter plot (Fig. 3). The scatter plot includes bubbles that represent individual countries and the average performance/score of three groups of countries, i.e. the Organisation of Economic Cooperation and Development (OECD), the Group of Twenty (G20) and the Least Developed Countries (LDC). The figure further shows that richer countries in general perform better on the SDG but are also associated with higher (i.e. worse) spillover effects internationally and have larger ecological footprints. The figure is for the year 2022 only. Additional illustrations for years 2019–2021 are available in Supplementary Information 2.

Illustrating the relationship between spillovers, economic development, and SDG performance, Fig. 3 highlights two salient trends: first, that economic development tends to displace negative environmental and social impacts from richer to poorer countries; and second, that SDG performance still closely tracks with conventional measures of development. If development displaces some negative environmental and social impacts from one country to another, it implies that countries ranking highly on SDSN’s SDG-Index ranking may be performing better than others on the SDGs because of the negative spillovers. It also suggests that they may be not fully accounting for the negative environmental and social impacts of their lifestyles and consumption due to the fact that some of those impacts are occurring in other countries.

## Discussion

Our regression results offer empirical evidence supporting the three hypotheses, providing a number of insights that are briefly discussed here. The correlation between the level of development and SDG performance in H1 could mean that richer countries are at a more favourable starting point to implement the SDGs. Richer countries usually have greater fiscal capacity and more effective governance policies, whether these initial conditions support the SDGs or not.

The positive correlation between economic development and SDG performance examined under H1 could also indicate that the measurement of SDG performance remains closely aligned with conventional business-as-usual that focuses more on economic dynamics. As such, methodologies to measure SDG progress may require adjustments going forward. Furthermore, the above-mentioned positive correlation could also imply that H1 is correct, thereby indicating a problem for SDG implementation: after nearly a decade of efforts, the SDGs have not been successful in triggering a significant transformation towards more sustainable societies^26^.

In H2 we examined the correlation between SDG performance and environmental impact. A positive relationship was also found here, which supports findings from other studies that countries tend to prioritise socio-economic dimensions over the environment^27–31^. At the same time, some countries also illustrate development patterns that are consistent with the Environmental Kuznets’ Curve (EKC)^32^. Once they reach a certain development threshold, their ecological footprint ‘decouples’ relative to economic performance^33^. While it may be true that environmental degradation slows down after a certain level of development, and countries can reduce some impacts by technological innovation or global trade, ultimately the planetary boundaries indicate an absolute limit to the tolerable impacts on ecosystems^34^, that if exceeded may hinder the achievement of the SDGs.

Trade is often referred to as an engine of growth and development, and thus provides further impetus to the richer countries. Data from the World Bank’s World Development Indicators^47^ reveal that high-income countries accounted for 64 percent of global merchandise trade in 2022. This share was 35 percent for low- and middle-income countries and just one percent for LDCs. While trade is essential for development, it can also be a double-edged sword in the sense that the impacts through trade may be displaced due to differences in labour force availability, wage rates and resource endowments. Accordingly, the associated negative environmental and social spillovers from trade-related economic activities can be a burden to exporting countries.

The third hypothesis (H3) examined the correlation between SDG performance and spillovers. Also, here the examination revealed a positive relationship, indicating that SDG progress may at least partly be a result of negative environmental and social impacts of consumption being displaced from one country to another. It is important to note that spillovers are an effect of a globalised economy, operating more based on profit motives rather than environmental or social indicators of success. Linking this finding with H2, it becomes clear that higher performing countries (per SDSN’s SDG Index) have not necessarily achieved absolute reduction of their environmental impacts but have ‘offshored’ them. According to the EKC, richer countries tend to pollute less (within their borders). However this does not explain why the total material footprint or the consumption-based ecological footprint tends to be highest in countries that should have already reached the flat part of the EKC curve^35^. In that sense, the EKC can only be observed on a scale that matches the economy, and if the economy (including its products, positive and negative impacts) has international links, then the evaluation of the performance should also reflect those links. Negative externalities cannot simply be hidden outside the domestic accounting system forever.

The findings presented in this paper can provide a starting point for discussions on important themes necessary for evaluating the entire SDG process and implementation. Such an evaluation is important for at least two reasons. First, a midpoint stocktake is vital now that the global community is more than halfway through the implementation period but nowhere close to meeting any of the 17 goals. Second, we are nearing 2030 and it is becoming increasingly relevant to evaluate the extent to which the SDGs measure sustainability or whether they remain aligned too closely with unsustainable growth. Any design of a follow-up agenda beyond 2030 would benefit from taking this into account.

Furthermore, it is clear that multi-disciplinary research is needed to tackle some of the limitations encountered in this study. One issue is that the concept of spillovers and the associated problems of displacing negative environmental and social impacts of development benefits is problematic for an agenda that strives to ‘Leave No one Behind’. This is especially the case if only a few countries could manage to achieve the SDGs by shifting the burden of their consumption onto other countries, thereby leaving other countries behind. To be sure, there are many difficult political and technical hurdles that need to be overcome before it will be possible to include spillovers in performance measurement^36^.

Key insights of this work relate particularly to the following two dimensions. First, regression analysis allows a comparison across a large number of data points and time series, providing a practical way to investigate larger correlational trends over time and space. Second, investigating the correlations between SDG performance and negative international impacts reveals broader trends on the international equity and burden-sharing of environmental benefits and burdens. This topic, while potentially difficult to discuss, is likely to gain traction as environmental impacts across the globe become increasingly evident, necessitating attention to these international linkages.

The spillovers issue illustrates the link between environmental and social issues as well as their international co-dependencies among countries. As such it is a good candidate to study the cooperation needed to enhance the equitable sharing of burdens and benefits of development. In addition to the prospects of international cooperation and synergies, there are policy implications in the areas of better accounting for international spillovers and reflecting vulnerabilities created by long and unsustainable value chains in pricing, so that consumers can make more informed purchasing and consumption choices. The range of policy options available or which need to be developed to address the spillovers issue are a subject for further research.

The question of sustainable development, and its progress thereof, is a global issue. Achieving progress in some countries while others lag behind will not put the world towards the path of sustainability. In this paper, we take a broad-based approach to show the close link between SDG performance and traditional economic growth metrics. We highlight how negative environmental and social effects from globally-sourced consumption act as barriers to achieving meaningful sustainability progress. Advantages of this the empirical method that we developed is that it allows comparison across a large data set revealing possible macro trends. At the same time, in using such method one loses a certain granularity that a closer study of a smaller sample size might reveal. We recommend follow-up studies to investigate the political and economic dynamics behind the occurrence of spillovers.

Hence, another area for future research work in this field could be to look at regional differences or clusters of suppliers and consumers of goods and services. Specific sectors such as critical minerals, garments or fashion could be examples for a deep dive on the international linkages and value chains, and the generated spillovers.

It will also be important to design practical approaches to allow such international spillovers to inform policymaking going forward so that benefits and impacts are distributed fairly across the lifecycle of products and services as they move across the world. Last but not least, SDG performance itself may not yet truly capture the environmental dimension of sustainability since high performing countries maintain unsustainably large environmental footprints. Therefore, it may be necessary to identify ways to measure sustainability performance that captures the importance of planetary health.

## Methods and data

We chose to use a panel data model for our regression since it enables combining cross-section analysis with time series. However, panel data models often tend to show heteroscedastic disturbances, i.e. the variance of the residuals is not constant across observations. Simple OLS models assume the variance to be constant, and thus the statistical tests of significance in OLS are likely to be invalid in the presence of heteroscedasticity. A well-known practice in the presence of heteroscedasticity is to use generalised least square (GLS) regression instead of ordinary least square (OLS)^37–39^. GLS models take into account that the cross-sections may have different characteristics. This is also feasible for our panel dataset which contains 163 cross-sections (i.e. countries; see Supplementary Information 3 for the list of countries.), each of which is different. Taking this into consideration, we used the panel estimated generalised least square (Panel EGLS) with cross-section random effect, as well as using EGLS with cross-section random effect and period fixed effect for the dummy variables.

### Model estimation and variables used

A linear regression can be developed to test the hypotheses and quantitatively demonstrate the relationship between SDG performance, international spillovers and economic prosperity using real-world data. The baseline specification is as follows (Eq. 1):

1 $$SDG\_Inde{x}_{it}= {\beta }_{0}+{\beta }_{1} GDP\_P{C\_PPP}_{it} +{\beta }_{2} Spillover\_Inde{x}_{it} +{\varepsilon }_{it}$$

where.

$${\beta }_{0}$$ = Unknown constant;

$SDG\_Inde{x}_{it}$ = SDG Index Score of country *i* at time *t,* measuring the country’s SDG performance;

$GDP\_{PC\_PPP}_{it}$ = Gross domestic product (GDP) per capita (in purchasing power parity [PPP] terms) of country *i* at time *t*, representing the country’s level of economic development (or richness);

$Spillover\_Inde{x}_{it}$ = Spillover Index Score of country *i* at time *t;* and ${\varepsilon }_{it}$ = Error term.

The SDG Index, taken from SDSN, is based on a number of assumptions of which the following are relevant to this paper. First, the 2030 Agenda evolves over time, meaning that data and measurement methodologies also improve over time as monitoring and measurement approaches improve to capture the intentions of the SDG targets. Similarly, even though country scores can be compared longitudinally, in the strictest sense the SDG Index results cannot be directly compared from one year to another. Second, where necessary, measurement is based on non-official data, sometimes using new data collection methods or forms of data that are not officially recognised. Third, the Index measures performance on a scale from 0 to 100, with a higher score indicating better performance. Although not perfect, it is currently the best available indicator that provides a comparable and consistent set of data on SDG performance for all countries. We chose this indicator as the dependent variable of our analysis.

The baseline estimation is then further extended to incorporate other key variables that impact sustainable development. First, we added a demographic variable as a control variable. Population dynamics significantly influence a country’s sustainable development efforts^40^. While demographic dividend is important, larger population size may overburden developmental activities. This variable is also an indication of the market size of a country, which is important in the discussions related to spillover effects. Next, we add a variable on a country’s engagement in international trade, with ample evidence suggesting that trade promotes economic growth and boosts poverty reduction and economic development^41–45^. The World Trade Organization (WTO) has also emphasised the role of trade in achieving the SDGs^46^. Furthermore, international spillovers, which form the central issue in our study, occur through international trade. Our test considers a country’s level of engagement in international trade, represented by total exports of the country.

Next, to incorporate the second hypothesis of our study, we looked into a country’s environmental resource use. The variable on ecological footprint of consumption within and beyond national boundaries assumes that SDG performance is positively linked with higher ecological footprint. Ecological footprint, as defined by the Global Footprint Network (GFN), ‘measures the ecological assets that a given population or product requires to produce the natural resources it consumes (including plant-based food and fibre products, livestock and fish products, timber and other forest products, space for urban infrastructure) and to absorb its waste, especially carbon emissions’^25^. Ecological Footprint of consumption is the sum of ecological footprint of production and net Ecological Footprint of trade (Ecological Footprint of imports minus Ecological Footprint of exports). The GFN data, reports ecological footprint of consumption as global hectares/capita, with disaggregation (built-up land, carbon, cropland, fishing grounds, forest products, and grazing land) and as total. In our study we used total ecological footprint of consumption per person.

To further explore the association between SDG performance and the economic affluence of the countries, we considered dummy variables for country groupings representing the level of affluence. For instance, the members of the Organisation for Economic Cooperation and Development (OECD) are among the most economically advanced and prosperous in the world. A country’s membership to OECD should thus indicate a positive impact on SDG performance relative to those countries which are not part of the OECD. We included three dummies to reflect Prosperity (OECD), Diversity (G20) and Poverty (LDC). These dummy variables are unity when a country is a member of any or more of these groupings, and zero otherwise.

With these specifications, the baseline estimation can be updated as follows (Eq. 2):

2 $$SDG\_Inde{x}_{it}= {\beta }_{0}+{\beta }_{1} log(POP{\_TOTAL}_{it})+{\beta }_{2} log(GDP\_{PC\_PPP}_{it})+{\beta }_{3} log(Exports)+{\beta }_{4} log(Ecological\_Footprin{t}_{it)}+{\beta }_{5} Spillover\_Inde{x}_{it}+{\beta }_{6} OEC{D}_{it}+{\beta }_{7} E{U}_{it}+{\beta }_{8} LD{C}_{it}+{\varepsilon }_{i}$$

where.

$${\beta }_{0}$$ = Unknown constant;

$SDG\_Inde{x}_{it}$ = SDG Index Score of country *i* at time *t,* measuring the country’s SDG performance;

$POP{\_TOTAL}_{it}$ = Total Population of country *i* at time *t*, representing the country’s demographic condition;

$GDP\_{PC\_PPP}_{it}$ = Gross domestic product (GDP) per capita (in PPP terms) of country *i* at time *t*, representing the country’s level of economic development (or richness);

${Exports}_{it}$ = Total exports of goods and services of country *i* at time t, representing the country’s trade engagement;

$Ecological\_Footprin{t}_{it}$ = Total ecological footprint of consumption per capita of country *i* at time t;

$OEC{D}_{it}$ = Dummy variable taking the value of 1 if country *i* is a member of the OECD at time *t*, 0 otherwise;

zero otherwise of country *i* at time t;

$E{U}_{it}$ = Dummy variable taking the value of 1 if country *i* is a member of the EU at time *t*, 0 otherwise;

$LD{C}_{it}$ = Dummy variable taking the value of 1 if country *i* is listed as one of the LDC countries at time *t*, 0 otherwise;

$Spillover\_Inde{x}_{it}$ = Spillover Index Score of country *i* at time t.

$${\varepsilon }_{it}$$ = Error term.

#### Data consistency

We checked if the data reflects normal distribution, or if there exists any skewness or kurtosis. This was done by developing a normal quantile-to-quantile (Q-Q) plot of the model residual, and we found that the data fit our model. For more information and the Q-Q plot, see Supplementary Information 4.

#### Data sources

Data for the dependent, explanatory and control variables have been collected from publicly available sources (Supplementary Information 5). The dependent variable, i.e. SDG performance, is represented by SDSN’s SDG Index Score^1^, which is a dimensionless index ranging from 0 to 100, with higher scores indicating better performance. Data for spillover is also collected from SDSN’s SDG Index database^1^. Like the SDG Index Score, the Spillover Index Score is also dimensionless where a country’s higher score indicates lesser negative spillover effects in other countries. GDP data (which represents a country’s level of economic development), and share of trade in GDP are collected from the World Bank’s World Development Indicators^47^. Dummy variables representing the three groups of countries are developed by the authors through a logical framework assessing if a given country was a member of a certain country group in one or more years during the covered time period. The dummy variables take the value of 1 when a country is part of a country group in a given year, and zero otherwise (Eq. 3).

3 $${\text{IF }}\left[ {\text{Country i}} \right]{\text{ IS IN }}\left[ {\text{Group r}} \right]{\text{ IN }}\left[ {\text{Year t}} \right],{\text{ THEN 1}}, \, 0$$

Data has been collected for 163 countries for the period 2000 to 2022. However, the Spillover Index Score is available only for four years (2019–2022). Thus, we used a panel dataset covering these four years for the 163 countries. While overall data availability for all the variables was quite good, there were some missing data points that drew our attention. Our data checking and exploration found missing data points at random for a single country for a given variable for one or more years, and on a few occasions, we found missing data for a country for a given variable for the whole data range. We also noticed that some of these missing data (both types) in key variables (e.g. Spillover Index) were mostly found in the developing and least developed countries. We assumed that this pattern of missing data, since it would reduce the number of observations in the model, would influence the regression results, and initial tests confirmed this. We applied data imputation techniques to overcome the problem. For variables where data for a given country was missing at random data points but was available for at least two data points, we imputed the missing data with the compound annual growth rate (CAGR) formula. The CAGR is a well-established technique that assumes that a given random variable “grows at a constant rate of return compounded over a sample period of time”^48^. Furthermore, when data was completely missing for a given variable for a country, we used proxy data from a similar country. For this, we looked into the Human Development Index (HDI) score from UNDP’s Human Development Report 2021–2022^49^ to select the proxy country. Thus, a balanced panel dataset with 163 cross-sections was used in the regression. See Supplementary Information 5 for the list of variables used in the regression.

### Supplementary Information

Supplementary Information 1.

Supplementary Information 2.

Supplementary Information 3.

Supplementary Information 4.

Supplementary Information 5.

## Data Availability

All data generated or analysed during this study are included in this published article (and its supplementary information files).
