# Author Correction: Examining the unsustainable relationship between SDG performance, ecological footprint and international spillovers

**DOI:** 10.1038/s41598-024-65254-3

**Published:** 2024-06-21

**Authors:** Mustafa Moinuddin, Simon Høiberg Olsen

**Affiliations:** Institute for Global Environmental Strategies, Hayama, Japan

Correction to: *Scientific Reports* 10.1038/s41598-024-61530-4, published online 17 May 2024

In the original version of this Article, the Acknowledgements section was omitted. It now reads,

**Acknowledgements**

"This research was supported by the Environment Research and Technology Development Fund [JPMEERF20221M03] of the Environmental Restoration and Conservation Agency provided by the Ministry of the Environment of Japan."

The original Article has been corrected.

