# Supplementary material for: Examining the unsustainable relationship between SDG performance, ecological footprint and international spillovers
Source: Sci Rep. 2024 May 17;14:11277. doi: 10.1038/s41598-024-61530-4 (PMC11101620; doi:10.1038/s41598-024-61530-4)
Supplement: Supplementary file 1 — Supplementary Information 1. [file 41598_2024_61530_MOESM1_ESM.docx]

**Supplementary Information 1**

Panel regression results

| Dependent Variable: SDG_INDEX | | |  |  |
| --- | --- | --- | --- | --- |
| Method: Panel EGLS (Cross-section random effects) | | | | |
| Date: 09/17/23 Time: 15:29 | | |  |  |
| Sample: 2019 2022 | | |  |  |
| Periods included: 4 | | |  |  |
| Cross-sections included: 160 | | |  |  |
| Total panel (balanced) observations: 640 | | | |  |
| Swamy and Arora estimator of component variances | | | | |
|  |  |  |  |  |
|  |  |  |  |  |
| Variable | Coefficient | Std. Error | t-Statistic | Prob. |
|  |  |  |  |  |
|  |  |  |  |  |
| LOG(POP_TOTAL) | -0.030295 | 0.291125 | -0.104062 | 0.9172 |
| LOG(GDP_PC_PPP) | 1.722472 | 0.343119 | 5.020043 | 0.0000 |
| LOG(EXPORTS) | 0.311664 | 0.146418 | 2.128599 | 0.0337 |
| LOG(ECOLOGICAL_FP) | 1.552825 | 0.406088 | 3.823865 | 0.0001 |
| SPILLOVER_INDEX | -0.011899 | 0.006040 | -1.970097 | 0.0493 |
| OECD | 0.638805 | 0.340017 | 1.878745 | 0.0607 |
| G20 | 1.083046 | 1.460219 | 0.741701 | 0.4585 |
| LDC | -9.982985 | 1.108278 | -9.007655 | 0.0000 |
| C | 45.73672 | 5.581805 | 8.193895 | 0.0000 |
|  |  |  |  |  |
|  |  |  |  |  |
|  | Effects Specification | |  |  |
|  |  |  | S.D. | Rho |
|  |  |  |  |  |
|  |  |  |  |  |
| Cross-section random | | | 4.789488 | 0.9905 |
| Period fixed (dummy variables) | | | |  |
| Idiosyncratic random | | | 0.469687 | 0.0095 |
|  |  |  |  |  |
|  |  |  |  |  |
|  | Weighted Statistics | |  |  |
|  |  |  |  |  |
|  |  |  |  |  |
| R-squared | 0.453758 | Mean dependent var | | 67.49764 |
| Adjusted R-squared | 0.444190 | S.D. dependent var | | 0.692395 |
| S.E. of regression | 0.516199 | Sum squared resid | | 167.3377 |
| F-statistic | 47.42484 | Durbin-Watson stat | | 1.247249 |
| Prob(F-statistic) | 0.000000 |  |  |  |
|  |  |  |  |  |
|  |  |  |  |  |
|  | Unweighted Statistics | |  |  |
|  |  |  |  |  |
|  |  |  |  |  |
| R-squared | 0.656926 | Mean dependent var | | 67.49764 |
| Sum squared resid | 22576.86 | Durbin-Watson stat | | 0.009244 |
|  |  |  |  |  |
|  |  |  |  |  |

Source: Authors, based on the modelling results.
