# Supplementary material for: Examining the unsustainable relationship between SDG performance, ecological footprint and international spillovers
Source: Sci Rep. 2024 May 17;14:11277. doi: 10.1038/s41598-024-61530-4 (PMC11101620; doi:10.1038/s41598-024-61530-4)
Supplement: Supplementary file 2 — Supplementary Information 2. [file 41598_2024_61530_MOESM2_ESM.docx]

**Supplementary Information 2**: Illustration of the relationship between SDG performance, spillover effect and ecological footprint (2019, 2020, 2021 and 2022)


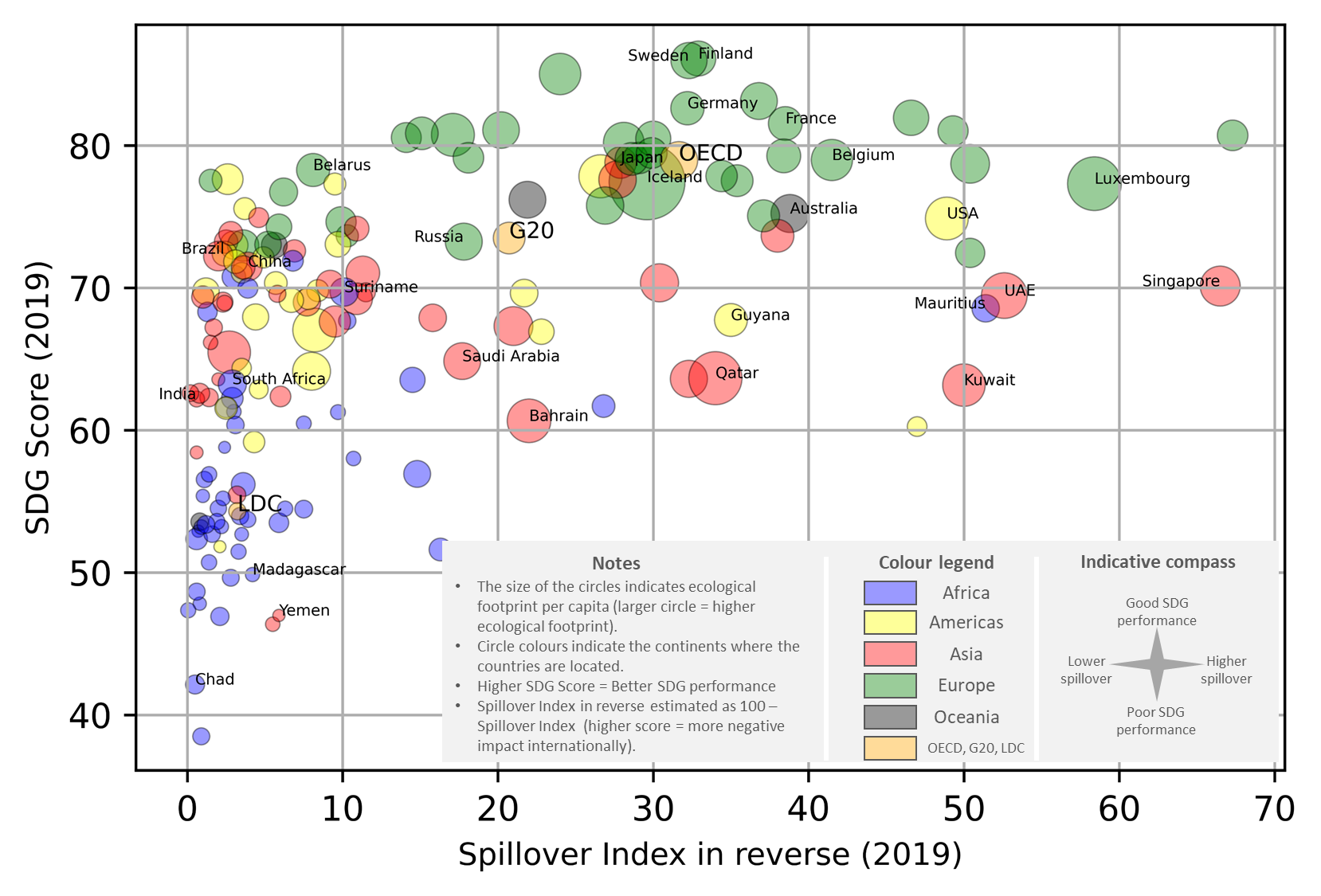


Source: Authors, based on data from SDSN and Global Footprint Network.


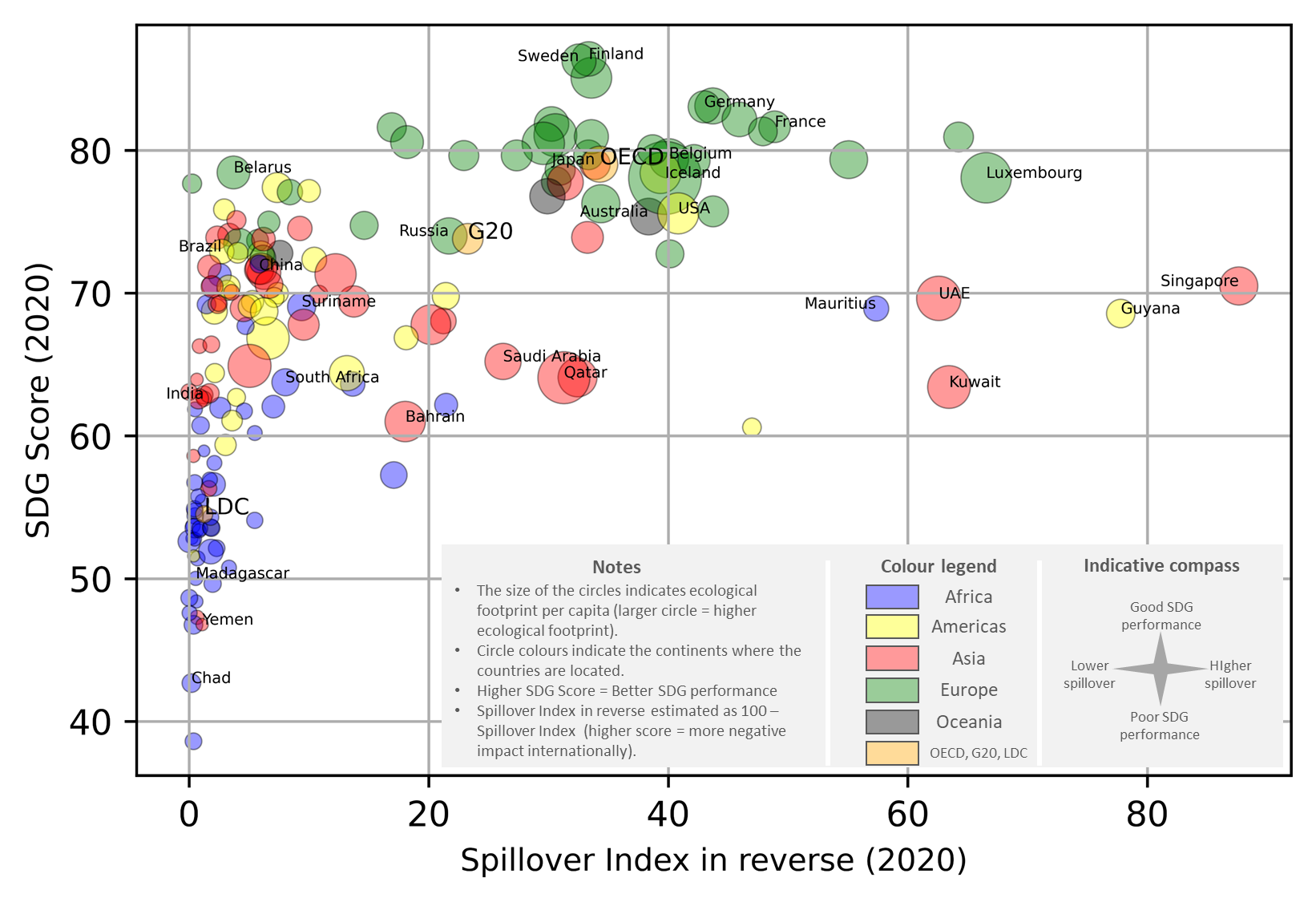


Source: Authors, based on data from SDSN and Global Footprint Network.


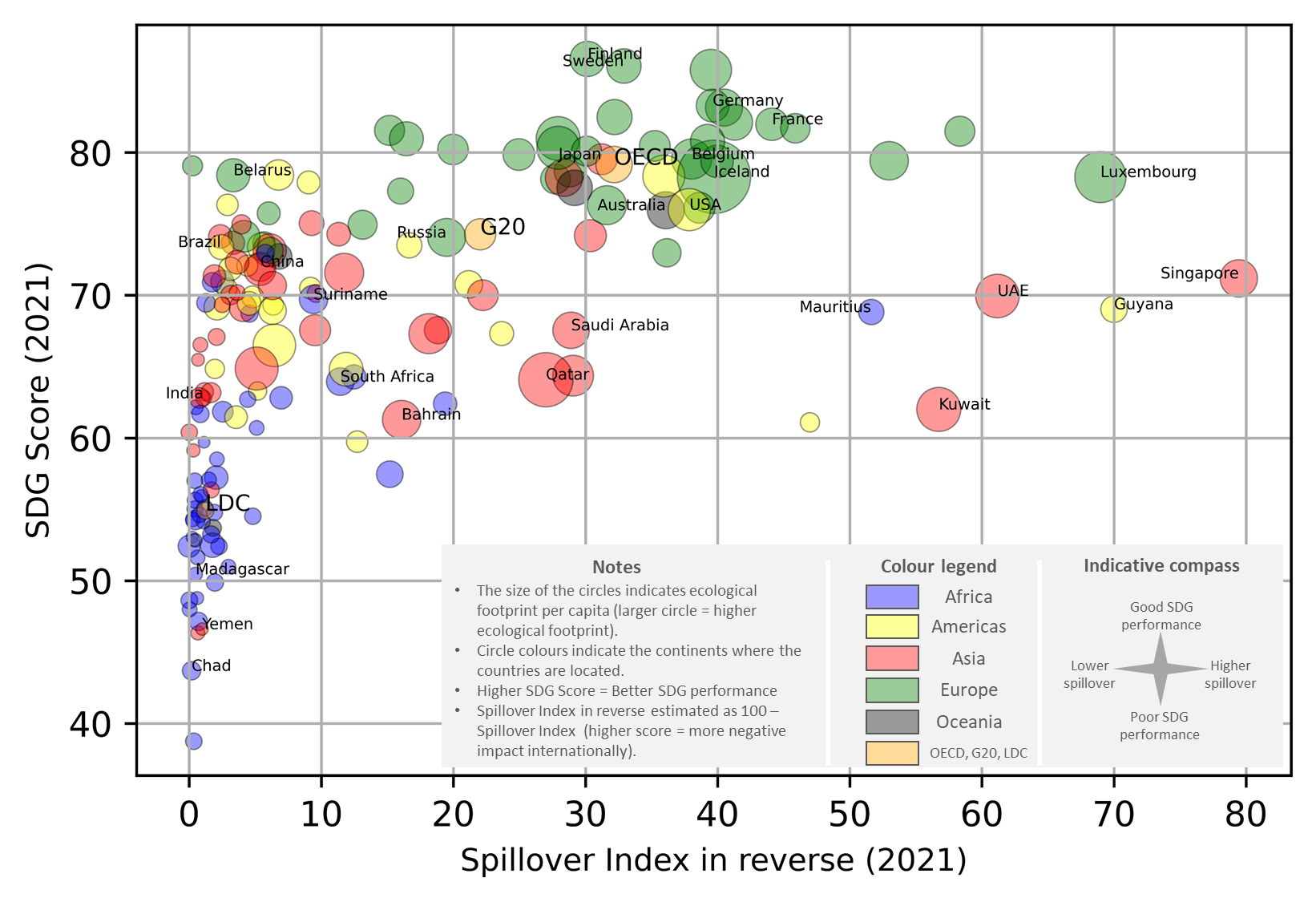


Source: Authors, based on data from SDSN and Global Footprint Network.


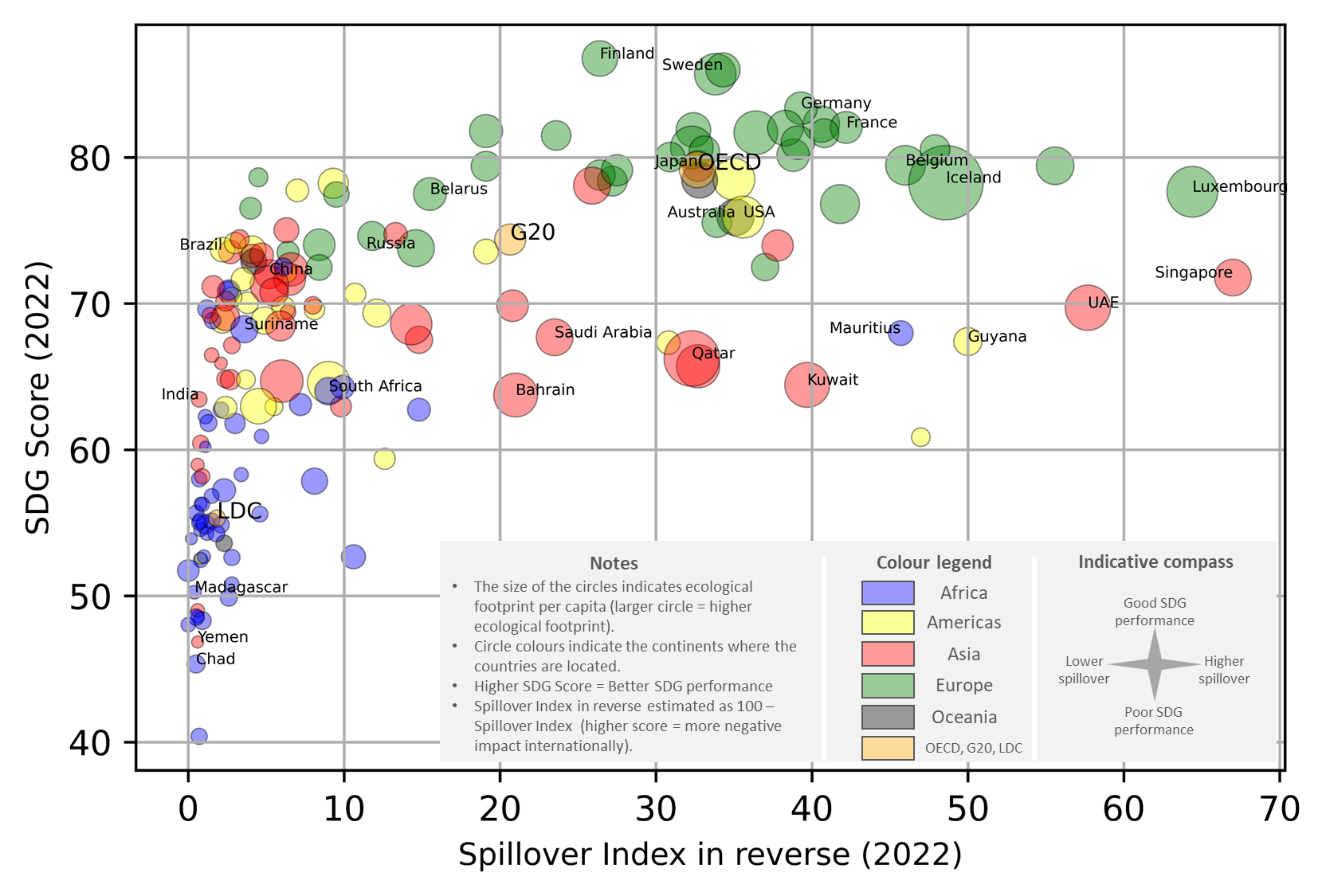


Source: Authors, based on data from SDSN and Global Footprint Network.
