# Supplementary material for: Examining the unsustainable relationship between SDG performance, ecological footprint and international spillovers
Source: Sci Rep. 2024 May 17;14:11277. doi: 10.1038/s41598-024-61530-4 (PMC11101620; doi:10.1038/s41598-024-61530-4)
Supplement: Supplementary file 3 — Supplementary Information 3. [file 41598_2024_61530_MOESM3_ESM.docx]

**Supplementary Information 3: List of countries included in the regression analysis (alphabetically)**

| 1 | Afghanistan |
| --- | --- |
| 2 | Albania |
| 3 | Algeria |
| 4 | Angola |
| 5 | Argentina |
| 6 | Armenia |
| 7 | Australia |
| 8 | Austria |
| 9 | Azerbaijan |
| 10 | Bahamas, The |
| 11 | Bahrain |
| 12 | Bangladesh |
| 13 | Barbados |
| 14 | Belarus |
| 15 | Belgium |
| 16 | Belize |
| 17 | Benin |
| 18 | Bhutan |
| 19 | Bolivia |
| 20 | Bosnia and Herzegovina |
| 21 | Botswana |
| 22 | Brazil |
| 23 | Brunei Darussalam |
| 24 | Bulgaria |
| 25 | Burkina Faso |
| 26 | Burundi |
| 27 | Cabo Verde |
| 28 | Cambodia |
| 29 | Cameroon |
| 30 | Canada |
| 31 | Central African Republic |
| 32 | Chad |
| 33 | Chile |
| 34 | China |
| 35 | Colombia |
| 36 | Comoros |
| 37 | Congo, Dem. Rep. |
| 38 | Congo, Rep. |
| 39 | Costa Rica |
| 40 | Cote d'Ivoire |
| 41 | Croatia |
| 42 | Cuba |
| 43 | Cyprus |
| 44 | Czech Republic |
| 45 | Denmark |
| 46 | Djibouti |
| 47 | Dominican Republic |
| 48 | Ecuador |
| 49 | Egypt, Arab Rep. |
| 50 | El Salvador |
| 51 | Estonia |
| 52 | Eswatini |
| 53 | Ethiopia |
| 54 | Fiji |
| 55 | Finland |
| 56 | France |
| 57 | Gabon |
| 58 | Gambia, The |
| 59 | Georgia |
| 60 | Germany |
| 61 | Ghana |
| 62 | Greece |
| 63 | Guatemala |
| 64 | Guinea |
| 65 | Guyana |
| 66 | Haiti |
| 67 | Honduras |
| 68 | Hungary |
| 69 | Iceland |
| 70 | India |
| 71 | Indonesia |
| 72 | Iran, Islamic Rep. |
| 73 | Iraq |
| 74 | Ireland |
| 75 | Israel |
| 76 | Italy |
| 77 | Jamaica |
| 78 | Japan |
| 79 | Jordan |
| 80 | Kazakhstan |
| 81 | Kenya |
| 82 | Korea, Rep. |
| 83 | Kuwait |
| 84 | Kyrgyz Republic |
| 85 | Lao PDR |
| 86 | Latvia |
| 87 | Lebanon |
| 88 | Lesotho |
| 89 | Liberia |
| 90 | Lithuania |
| 91 | Luxembourg |
| 92 | Madagascar |
| 93 | Malawi |
| 94 | Malaysia |
| 95 | Maldives |
| 96 | Mali |
| 97 | Malta |
| 98 | Mauritania |
| 99 | Mauritius |
| 100 | Mexico |
| 101 | Moldova |
| 102 | Mongolia |
| 103 | Morocco |
| 104 | Mozambique |
| 105 | Myanmar |
| 106 | Namibia |
| 107 | Nepal |
| 108 | Netherlands |
| 109 | New Zealand |
| 110 | Nicaragua |
| 111 | Niger |
| 112 | Nigeria |
| 113 | North Macedonia |
| 114 | Norway |
| 115 | Oman |
| 116 | Pakistan |
| 117 | Panama |
| 118 | Papua New Guinea |
| 119 | Paraguay |
| 120 | Peru |
| 121 | Philippines |
| 122 | Poland |
| 123 | Portugal |
| 124 | Qatar |
| 125 | Romania |
| 126 | Russian Federation |
| 127 | Rwanda |
| 128 | Sao Tome and Principe |
| 129 | Saudi Arabia |
| 130 | Senegal |
| 131 | Sierra Leone |
| 132 | Singapore |
| 133 | Slovak Republic |
| 134 | Slovenia |
| 135 | Somalia |
| 136 | South Africa |
| 137 | Spain |
| 138 | Sri Lanka |
| 139 | Sudan |
| 140 | Suriname |
| 141 | Sweden |
| 142 | Switzerland |
| 143 | Syrian Arab Republic |
| 144 | Tajikistan |
| 145 | Tanzania |
| 146 | Thailand |
| 147 | Togo |
| 148 | Trinidad and Tobago |
| 149 | Tunisia |
| 150 | Turkiye |
| 151 | Turkmenistan |
| 152 | Uganda |
| 153 | Ukraine |
| 154 | United Arab Emirates |
| 155 | United Kingdom |
| 156 | United States |
| 157 | Uruguay |
| 158 | Uzbekistan |
| 159 | Venezuela, RB |
| 160 | Vietnam |
| 161 | Yemen, Rep. |
| 162 | Zambia |
| 163 | Zimbabwe |
