# Supplementary material for: Examining the unsustainable relationship between SDG performance, ecological footprint and international spillovers
Source: Sci Rep. 2024 May 17;14:11277. doi: 10.1038/s41598-024-61530-4 (PMC11101620; doi:10.1038/s41598-024-61530-4)
Supplement: Supplementary file 4 — Supplementary Information 4. [file 41598_2024_61530_MOESM4_ESM.docx]

**Supplementary Information 4**: Data consistency check

We checked the distribution by first deriving the model residual (RESID) and then developing a normal quantile-to-quantile (Q-Q) plot of RESID (on the horizontal axis) against normal (on the vertical axis) (See the figure below). The resulting points displayed almost perfect linearity indicating that the data is normally distributed, with limited departures from normality at the top and the bottom. Put differently, based on the Q-Q plot visual check, we can suggest that the data fit, for the most part, our regression model.

Figure Q-Q plot of the residual

Source: Authors’ generated.
