# Supplementary material for: Examining the unsustainable relationship between SDG performance, ecological footprint and international spillovers
Source: Sci Rep. 2024 May 17;14:11277. doi: 10.1038/s41598-024-61530-4 (PMC11101620; doi:10.1038/s41598-024-61530-4)
Supplement: Supplementary file 5 — Supplementary Information 5. [file 41598_2024_61530_MOESM5_ESM.docx]

**Supplementary information 5**

List of variables used in the regression

| **Type of variable** | **Theme** | **Indicator used** | **Unit** | **Log transformation** | **Data source** |
| --- | --- | --- | --- | --- | --- |
| Dependent | SDG performance | SDSN’s SDG Index Score | Dimensionless index (0 – 100) | No | Homepage of Sustainable Development Solutions Network  Excel Database file. Available at:  https://dashboards.sdgindex.org/downloads |
| Explanatory | Spillover effect | SDSN’s Spillover Index score | Dimensionless index (0 – 100) | No | Homepage of Sustainable Development Solutions Network  Excel Database file. Available at:  https://dashboards.sdgindex.org/downloads |
| Explanatory | Environmental footprint | Ecological footprint of consumption per person | Global hectares | Yes | Data Portal of Global Foodprints Network  https://data.footprintnetwork.org/#/ |
| Control | Demography | Total population | Number of people | Yes | World Bank’s World Development Indicators Database  https://databank.worldbank.org/source/world-development-indicators |
| Control | Economic development | GDP Per Capita (PPP) | Constant 2017 international dollar | Yes | World Bank’s World Development Indicators Database  https://databank.worldbank.org/source/world-development-indicators |
| Control | Trade engagement | Total exports | Current US dollar | Yes | World Bank’s World Development Indicators Database  https://databank.worldbank.org/source/world-development-indicators |
| Dummy | Prosperity (OECD); | Country membership to OECD | Binary (1 or 0) | No | Based on a logical framework developed by the authors and using information from OECD Homepage  https://www.oecd.org/about/document/ratification-oecd-convention.htm |
| Dummy | Diversity (G20) | Country membership to G20 | Binary (1 or 0) | No | Based on a logical framework developed by the authors and using information from G20 Homepage  http://g20.org.tr/about-g20/g20-members/index.html |
| Dummy | Poverty (LDC) | Inclusion in the list of LDC countries | Binary (1 or 0) | No | Based on a logical framework developed by the authors and using information from United Nations Homepage  https://www.un.org/ohrlls/content/list-ldcs |

Source: Authors
